# Highly Efficient, Low-Cost, and Magnetically Recoverable FePt–Ag Nanocatalysts: Towards Green Reduction of Organic Dyes

**DOI:** 10.3390/nano8050329

**Published:** 2018-05-14

**Authors:** Yang Liu, Yuanyuan Zhang, Qiangwei Kou, Yue Chen, Yantao Sun, Donglai Han, Dandan Wang, Ziyang Lu, Lei Chen, Jinghai Yang, Scott Guozhong Xing

**Affiliations:** 1 College of Physics, Jilin Normal University, Siping 136000, China; liuyang@jlnu.edu.cn (Y.L.); 13944139606@163.com (Y.Z.); 13944949603@163.com (Q.K.); 17649973053@163.com (Y.C.); chenlei@jlnu.edu.cn (L.C.); 2 Key Laboratory of Functional Materials Physics and Chemistry of the Ministry of Education, Jilin Normal University, Changchun 130103, China; syt@jlnu.edu.cn; 3 School of Materials Science and Engineering, Changchun University of Science and Technology, Changchun 130022, China; dlhan_1015@cust.edu.cn; 4 Technology Development Department, GLOBALFOUNDRIES (Singapore) Pte. Ltd., 60 Woodlands Industrial Park D, Street 2, Singapore 738406, Singapore; DANDAN.WANG@globalfoundries.com; 5 School of Environment and Safety Engineering, Jiangsu University, Zhenjiang 212013, China; lzy@mail.ujs.edu.cn; 6 United Microelect Corp. Ltd., 3 Pasir Ris Dr 12, Singapore 519528, Singapore

**Keywords:** FePt–Ag nanocomposites, magnetic properties, catalytic reduction, organic dyes

## Abstract

Nowadays, synthetic organic dyes and pigments discharged from numerous industries are causing unprecedentedly severe water environmental pollution, and conventional water treatment processes are hindered due to the corresponding sophisticated aromatic structures, hydrophilic nature, and high stability against light, temperature, etc. Herein, we report an efficient fabrication strategy to develop a new type of highly efficient, low-cost, and magnetically recoverable nanocatalyst, i.e., FePt–Ag nanocomposites, for the reduction of methyl orange (MO) and rhodamine B (RhB), by a facile seed deposition process. X-ray diffraction results elaborate that the as-synthesized FePt–Ag nanocomposites are pure disordered face-centered cubic phase. Transmission electron microscopy studies demonstrate that the amount of Ag seeds deposited onto the surfaces of FePt nanocrystals increases when increasing the additive amount of silver colloids. The linear correlation of the MO and RhB concentration versus reaction time catalyzed by FePt–Ag nanocatalysts is in line with pseudo-first-order kinetics. The reduction rate constants of MO and RhB increase with the increase of the amount of Ag seeds. FePt–Ag nanocomposites show good separation ability and reusability, and could be repeatedly applied for nearly complete reduction of MO and RhB for at least six successive cycles. Such cost-effective and recyclable nanocatalysts provide a new material family for use in environmental protection applications.

## 1. Introduction

At present, water environmental pollution has become a major global issue. Excessive emissions of the dye effluents derived from the textile, leather, food, paper, plastic, rubber, and pharmaceutical industries are one of the most important water pollution sources [1,2,3,4]. These dispersed dye molecules block sunlight from reaching the lower depths of the water, reduce the dissolved oxygen level in the water, and thereby inhibit the growth and development of marine plants and animals [5,6,7]. Most seriously, most of these dyes are mutagenic and carcinogenic and cause severe damage to human life [8,9,10]. Therefore, it is necessary to remove the dyes from wastewater for environmental protection and remediation. Unfortunately, it is hard to treat organic dye contaminants because their structures are stable and biologically nondegradable [11,12].

Conventional treatment techniques to remove and decolorize organic dyes include adsorption, biodegradation, ultrafiltration, photochemical, and electrochemical methods [13]. Due to the constraints like the complex aromatic structure and recalcitrant nature of the dyes or the high operating cost, the above techniques are difficult to popularize in practical applications. Recently, photocatalytic degradation in the presence of semiconductor photocatalysts (e.g., typically, TiO_2_ and ZnO) and catalytic reduction of noble metal catalysts (e.g., typically, Ag and Au) have developed as alternatives to conventional water treatment methods for the treatment of organic dyes [14,15,16]. For instance, Li et al. synthesized TiO_2_ to catalyze and degrade methyl orange (MO) under UV light irradiation [17]. However, even though the photocatalytic method is cost effective, the photocatalytic efficiency is low and not effective enough for the dye degradation. Another disadvantage is that the electrons and the holes of the semiconductor photocatalysts can only be excited under ultraviolet light irradiation.

Most recently, the catalytic reduction method for the removal of dyes by using noble metal nanocatalysts has been regarded as a competitive technology because of the high efficiency and simplicity of its design and operation. It must be mentioned that the noble metal nanocatalysts encounter an obstacle because they are difficult to efficiently separate and recycle after the catalytic process by traditional methods such as centrifugation or filtration. To address this problem, many researchers have tried to immobilize the nanocatalysts onto magnetic supporting materials [18,19,20,21]. Zhang et al. synthesized Fe_3_O_4_/Ag composites to catalyze rhodamine B (RhB) [22]. Bimetallic FePt nanocrystals have high chemical stability and fast magnetic responsiveness [23]. Interestingly, research results have found that FePt nanocrystals themselves have catalytic degradation abilities [24,25]. Therefore, considerable efforts have been devoted to the design and preparation of FePt–Ag nanocrystals for the degradation of dyes.

**Figure 1:**
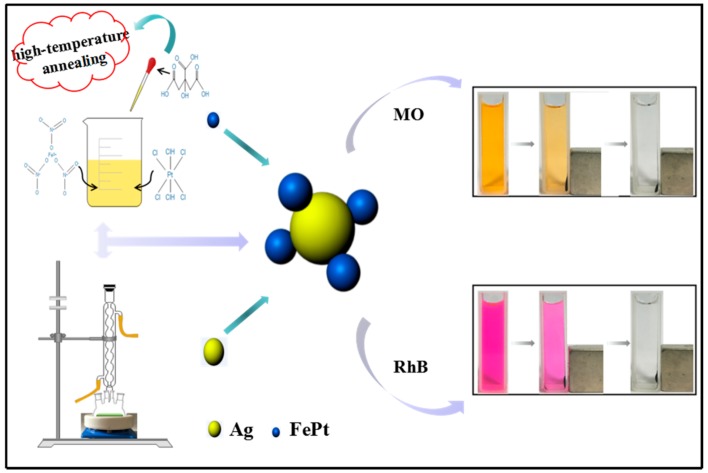
Schematic illustration of the synthesis and application of FePt–Ag nanocomposites.

In this work, with the aim of ensuring the ability of the proposed catalytic degradation system not only for a specific dye but also for other industrial dyes, methyl orange (MO) and rhodamine B (RhB) were selected as representative model dye pollutants. Polyethyleneimine dithiocarbamate (PEI-DTC) was employed as a polymer to prepare FePt–Ag nanocomposites by the seed deposition process. By adjusting the additive amount of silver colloids or the additive amount of FePt nanocrystals, we control the amount of Ag seeds deposited on the modified surfaces of FePt nanocrystals. In addition, in order to investigate the catalytic ability of FePt–Ag nanocomposites, we investigate the effects of the amount of Ag seeds on the catalytic capacities of the catalytic reduction of MO and RhB with the help of sodium borohydride (NaBH_4_). A schematic illustration of the synthesis and application of FePt–Ag nanocomposites is shown in Figure 1.

## 2. Experimental

### 2.1. Materials

The initial materials included iron (III) nitrate nonahydrate (Fe(NO_3_)_3_·9H_2_O), chloroplatinic acid hexahydrate (H_2_PtCl_6_·6H_2_O), citric acid monohydrate (C_6_H_8_O_7_·H_2_O), polyethyleneimine (PEI, branched, MW ≈ 25,000 g/mol), potassium hydroxide (KOH), carbon disulfide (CS_2_), silver nitrate (AgNO_3_), and trisodium citrate dihydrate (C_6_H_5_Na_3_O_7_·2H_2_O). All chemical reagents in this work were used as received without further purification.

### 2.2. Preparation of Silver Colloids

Noble metal nanocrystals have received both fundamental and practical attention owing to their potential applications in many fields such as ultrasensitive biosensing, imaging agents, photothermal therapy, and in particular catalysts because of their unique and tunable optical properties and high catalytic activities [26,27,28,29,30,31,32,33]. In the present work, the silver colloids were prepared based on the work by Yang et al. [34]. Briefly, 36 mg of AgNO_3_ was dissolved in 200 mL of deionized water under vigorous stirring at 90 °C. The solution was heated to near boiling after 15 min, and then 40 mg of 4 mL C_6_H_5_Na_3_O_7_·2H_2_O was injected into the solution drop by drop. The temperature of the solution was immediately reduced to 85 °C and the mixture was stirred continuously for another 40 min. Finally, the reaction mixture was allowed to cool down to room temperature and stored in the dark.

### 2.3. Preparation of Ag@PEI-DTC Solution

This process included two steps. The first step was the synthesis of PEI-DTC. Quantities of 50 mg of PEI and 325 mg of KOH were dissolved in 25 mL methanol. After the mixture solution was degassed, 347.5 μL of CS_2_ was added dropwise to the mixture solution; PEI-DTC aqueous solution was thus obtained. The second step was the synthesis of Ag@PEI-DTC solution. A quantity of 60mL of silver colloids was centrifuged and then dispersed in 20 mL of methanol. The obtained silver solution was added into the PEI-DTC solution drop by drop. After the reaction mixture was kept at room temperature for 1 h, the resultant product was collected by centrifugation (5000 rpm, 10 min) and subsequently washed with deionized water and ethanol several times. Finally, the obtained Ag@PEI-DTC seeds were stored in 4 mL of deionized water.

### 2.4. Preparation of FePt Nanocrystals

The FePt nanocrystals were synthesized using the sol-gel method. Details of the preparation have been published in our previous work [35]. In general, 234 mg of Fe(NO_3_)_3_·9H_2_O, 20 mL of H_2_PtCl_6_·6H_2_O, and 162 mg of C_6_H_8_O_7_·H_2_O were dissolved in 50 mL of deionized water with stirring to form the sol. The solution was polymerized to form a gel at 80 °C. FePt nanocrystals were successfully synthesized with precursors heat-treated in argon atmosphere for 2 h at 400 °C with a heating rate of 2 °C min^−1^.

### 2.5. Preparation of FePt–Ag@PEI-DTC Nanocomposites

Most recently, considerable efforts have been devoted to the design and preparation of multifunctional nanocomposites. Compared to the bulk materials, nanostructured materials possess large surface area while retaining their excellent electrical, optical, and magnetic properties [36,37,38,39,40,41,42,43]. A quantity of 10 mg of FePt nanocrystals was dispersed in 4 mL of deionized water and then added dropwise into the obtained Ag@PEI-DTC solution under vortex. FePt–Ag@PEI-DTC nanocomposites were shaken for 1 h and then washed with deionized water and ethanol three times; the result was named FePt–Ag 10 mg–60 mL. The experiment was carried out again under the same conditions as FePt–Ag 10 mg–60 mL, but the additive amount of silver colloids was adjusted to 90 and 120 mL, with the results named FePt–Ag 10 mg–90 mL and FePt–Ag 10 mg–120 mL, respectively. In addition, in order to study the effect of the additive amount of FePt nanocrystals on the amount of Ag seeds deposited on the modified surfaces of FePt nanocrystals, the volume of the silver colloids was kept the same as in FePt–Ag 10 mg–60 mL, but the mass of FePt nanocrystals was changed to 5 mg; the result was named FePt–Ag 5 mg–60 mL.

### 2.6. Application of FePt–Ag Nanocomposites for Catalytic Reduction of MO and RhB

The catalytic reduction of MO and RhB in the presence of NaBH_4_ was studied and monitored by a UV–vis spectroscopy to explore the catalytic activity of FePt–Ag 10 mg–120 mL. The detailed reaction process was as follows: 1 mg of FePt–Ag 10 mg–120 mL was added to 2 mL of MO or RhB aqueous solution (40 mg·L^−1^). Subsequently, 0.1 mL of NaBH_4_ aqueous solution (0.1 M, freshly prepared each time before use) was injected into the mixture solution. UV–vis spectra of the solution were recorded every 6 min. The first obtained data were designated as the values for the reaction time *t* = 0. The rate constant of the reduction process was determined by measuring the change in absorption as a function of time. It could be observed that the solution color changed gradually from colored to colorless. In order to investigate the reusability of the prepared samples, the FePt–Ag 10 mg–120 mL nanocatalysts were separated from the reaction solution using a magnet after the completion of the reduction process. The obtained magnetic nanocatalysts were washed with ethanol and deionized water three times and reused in the next reaction process. The same procedure for the catalytic reduction of each of MO and RhB was repeated six times.

### 2.7. Characterization Methods

Structure characterization of the as-synthesized samples was carried out on a Rigaku D/Max-2500 copper (Rigaku Corporation, Tokyo, Japan) rotating-anode X-ray diffractometer (XRD) using Cu Kα radiation (40 kV, 200 mA). Ultraviolet–visible spectroscopy (UV–vis) absorbance spectra were recorded on a Shimadzu UV 3600 spectrophotometer (Shimadzu Corporation, Tokyo, Japan) in the range of 350–800 nm. The detailed structure of the obtained samples was characterized using a JEOL JSM-7800F field emission scanning electron microscope (FESEM) (JEOL Ltd., Tokyo, Japan) at an acceleration voltage of 20 kV and a JEOL 2100 transmission electron microscope (TEM) operating at 200 kV. The X-ray photoelectron spectrum was measured with a Thermo Scientific ESCALAB 250Xi X-ray photoelectron spectroscope (XPS) (Thermo Fisher Scientific, Waltham, MA, USA). Magnetic properties were measured using a Quantum Design MPMS3 superconducting quantum interference device (SQUID) magnetometer (Quantum Design, Inc., San Diego, CA, USA).

## 3. Results and Discussion

### 3.1. Structure of FePt–Ag Nanocomposites

**Figure 2:**
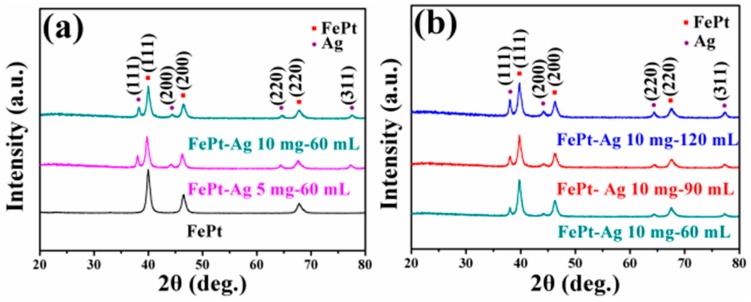
XRD patterns of the pure FePt nanocrystals and the FePt–Ag nanocomposites with different additive quantities of FePt nanocrystals (FePt–Ag 5 mg–60 mL and FePt–Ag 10 mg–60 mL) (**a**), and of the FePt–Ag nanocomposites with different additive amounts of silver colloids (FePt–Ag 10 mg–60 mL, FePt–Ag 10 mg–90 mL, and FePt–Ag 10 mg–120 mL) (**b**).

The crystal structure of pure FePt and FePt–Ag nanocomposites was investigated using XRD. Figure 2a,b present XRD patterns of the pure FePt nanocrystals and the FePt–Ag nanocomposites with different additive amounts of FePt nanocrystals (FePt–Ag 5 mg–60 mL and FePt–Ag 10 mg–60 mL), and of the FePt–Ag nanocomposites with different additive amounts of silver colloids (FePt–Ag 10 mg–60 mL, FePt–Ag 10 mg–90 mL, and FePt–Ag 10 mg–120 mL), respectively. As for the pure FePt nanocrystals, three primary peaks of (111), (200), and (220) around 39.8°, 46.4°, and 67.6° are observed and no other trace of impurities are detected, indicating that the as-synthesized FePt nanocrystals are pure disordered face-centered cubic phase [44]. After silver colloids are added to FePt nanocrystals, three additional diffraction peaks at about 38.3°, 44.5°, and 64.7° appear; these can be well attributed to the (111), (200), and (220) of Ag (Joint Committee on Powder Diffraction Standards, JCPDS card no. 04-0783) [45], respectively. With the decrease of the additive amount of FePt nanocrystals or the increase of the additive amount of silver colloids, the intensity of Ag diffraction peaks is obviously enhanced, but the position of Ag diffraction peaks does not change significantly. On the basis of these XRD results, we preliminarily conclude that the number of Ag seeds attached on the FePt surfaces can be adjusted by changing the additive amount of silver colloids or FePt nanocrystals.

**Figure 3:**
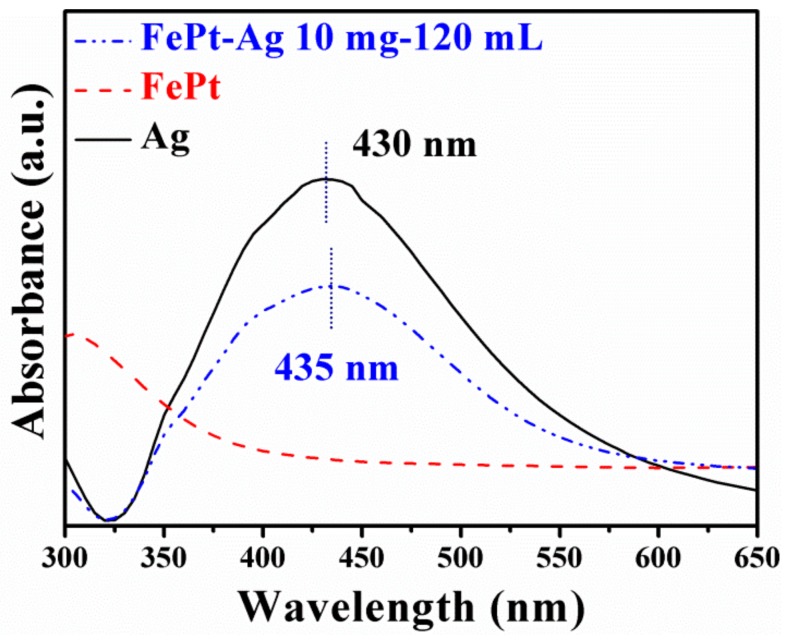
UV–vis absorbance spectra of silver colloids (black curve line), pure FePt nanocrystals (red dotted line), and FePt–Ag 10 mg–120 mL nanocomposites (blue dotted line).

UV–vis absorbance spectra were used to further confirm that the Ag seeds were firmly immobilized on the surfaces of FePt nanocrystals. Figure 3 shows the UV–vis absorbance spectra of silver colloids, FePt nanocrystals, and FePt–Ag 10 mg–120 mL nanocomposites. It is well established that silver colloids exhibit a plasmon absorption band in the visible region on account of the coherent excitation of the free electrons within the conduction band [46]. In general, the plasmon absorption band of silver nanocrystals is around 420 nm, while Fe and Pt nanocrystals have no specific absorption peak in this region [47]. As can be seen from Figure 3, the as-prepared silver colloids have a characteristic absorption peak at 430 nm, but no such absorption peak is observed for the FePt nanocrystals. An interesting finding is that the absorption peak of FePt–Ag 10 mg–120 mL shows a slight red shift from 430 to 435 nm compared with that of the silver colloids, which might be attributed to the polarizability of the metallic silver owing to the formation of the FePt–Ag nanocomposites [48].

### 3.2. Morphology of FePt–Ag Nanocomposites

**Figure 4:**
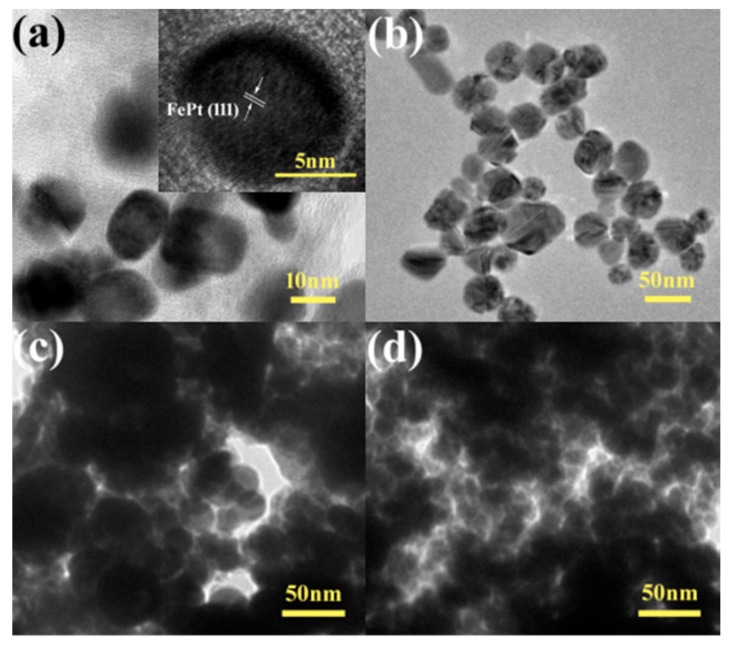
TEM images of the pure FePt nanocrystals with high-resolution TEM (HRTEM) image (inset) (**a**), pure silver colloids (**b**), and FePt–Ag 5 mg–60 mL (**c**) and FePt–Ag 10 mg–60 mL nanocomposites (**d**).

**Figure 5:**
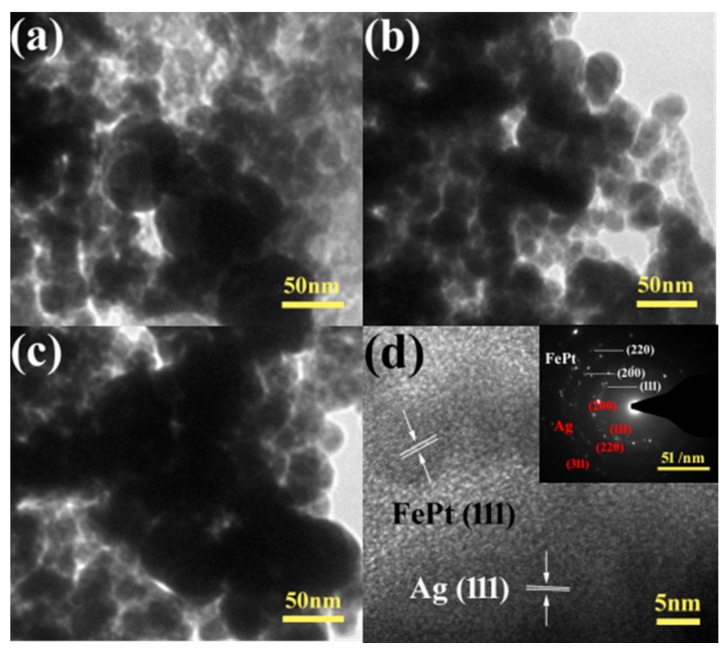
TEM images of FePt–Ag 10 mg–60 mL (**a**), FePt–Ag 10 mg–90 mL (**b**), and FePt–Ag 10 mg–120 mL (**c**); (**d**) shows the corresponding selected area electron diffraction (SAED) pattern (inset) and HRTEM image of (**c**).

The morphology and detailed structure of the samples were investigated by SEM and TEM. In particular, the TEM technique is a direct and important methodology for structure analysis in the materials engineering technologies [49,50,51,52,53,54,55,56,57,58,59,60,61]. Figure 4 shows typical low-magnification TEM images of pure FePt nanocrystals, pure silver colloids, and FePt–Ag 5 mg–60 mL and FePt–Ag 10 mg–60 mL nanocomposites. The shapes of both the pure FePt nanocrystals and silver colloids are nearly spherical, and their average particle sizes are 11 ± 1.5 and 41 ± 2 nm, respectively. The corresponding particle size histogram of the pure FePt nanocrystals indicates that FePt nanocrystals have a narrow particle size distribution (Figure S1). The inset of Figure 4a is the corresponding high-resolution TEM (HRTEM) image, which shows the characteristic spacings of 0.223 nm for the (111) lattice planes of face-centered cubic FePt. From the TEM images of FePt–Ag 5 mg–60 mL and FePt–Ag 10 mg–60 mL, Ag seeds are found to be coherent with FePt nanocrystals owing to the effect of the two chelating sulfur groups provided by the PEI-DTC polymers. It is commonly known that the brightness in the TEM image reflects the ability of the transmitted electrons from the different substances and is proportional to the atomic number of the substances [62]. Unfortunately, because both FePt and Ag nanocrystals have high atomic numbers, it is hard to distinguish the two nanomaterials from TEM images. TEM images of the FePt nanocrystals with different addition quantities of the silver colloids (FePt–Ag 10 mg–60 mL, FePt–Ag 10 mg–90 mL, and FePt–Ag 10 mg–120 mL) are exhibited in Figure 5. In order to investigate the influence of the additive amount of silver colloids on the number of Ag seeds attached to the FePt surfaces, energy-dispersive spectroscopy (EDS) was performed to determine the composition of the nanostructures, as shown in Figure S2. The EDS results confirm that FePt–Ag nanocomposites are composed of Fe, Pt, and Ag, and that the amount of Ag seeds adhered to the surfaces of FePt nanocrystals increases with the increase in the additive amount of silver colloids. This is consistent with the observation from XRD results. The Si peak could be assigned to the Si substrates for loading the samples. HRTEM images show that the interplanar spacings of 0.223 and 0.235 nm match well with those of the (111) planes of FePt and Ag, respectively. The selected area electron diffraction (SAED) pattern of FePt–Ag 10 mg–120 mL is presented in the inset of Figure 5d. The bright and discrete diffraction spots can be assigned to the diffractions of either FePt nanocrystals or Ag seeds. HRTEM and SAED results further testify the coexistence of FePt and Ag.

### 3.3. XPS Studies of FePt–Ag Nanocomposites

**Figure 6:**
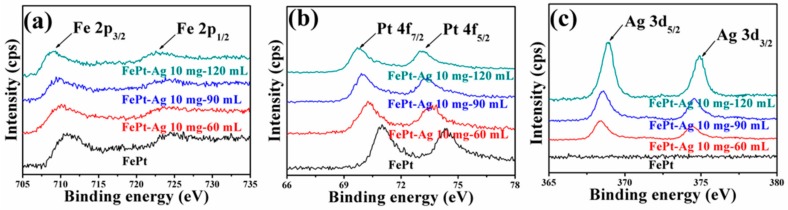
High-resolution XPS scans of Fe *2p* (**a**), Pt *4f* (**b**). and Ag *3d* (**c**) for pure FePt nanocrystals and for FePt–Ag 10 mg–60 mL, FePt–Ag 10 mg–90 mL, and FePt–Ag 10 mg–120 mL nanocomposites.

XPS measurements were carried out to characterize the chemical state of Fe, Pt, and Ag elements. High-resolution scans of Fe *2p*, Pt *4f*, and Ag *3d* for pure FePt nanocrystals and for FePt–Ag 10 mg–60 mL, FePt–Ag 10 mg–90 mL, and FePt–Ag 10 mg–120 mL nanocomposites are shown in Figure 6. As for the pure FePt nanocrystals, the Fe *2p* spectrum shows two contributions—*2p_3/2_* and *2p_1/2_*—resulting from the spin–orbit splitting, respectively located at 710.6 and 724.1 eV. The XPS spectrum of Pt exhibits double peaks with binding energies of 71.1 and 74.2 eV, corresponding to the standard peaks of pure Pt *4f_7/2_* and Pt *4f_5/2_* [63,64]. The binding energy of FePt–Ag 10 mg–60 mL is located at 367.4 and 373.4 eV, which can be assigned to Ag *3d_5/2_* and Ag *3d_3/2_*, and matches well with the standard XPS spectrum of metallic Ag [53,54]. It should be pointed out that the intensity of Fe *2p* and Pt *4f* decreases and the intensity of Ag *3d* increases with the increase of the additive amount of the silver colloids, since the intensity of XPS spectra is proportional to the atomic concentration [65]. Another interesting point is that the positions of Fe *2p* and Pt *4f* peaks shift toward the lower binding energy side, and the positions of Ag *3d* peaks shift toward the higher binding energy side. The shift of the binding energy provides evidence of an electronic exchange interaction between FePt and Ag [66]. Furthermore, in addition to Fe, Pt, Ag, O, and C peaks, no impurities are observed from the XPS survey scan spectra in Figure S3.

### 3.4. Magnetism of FePt–Ag Nanocomposites

**Figure 7:**
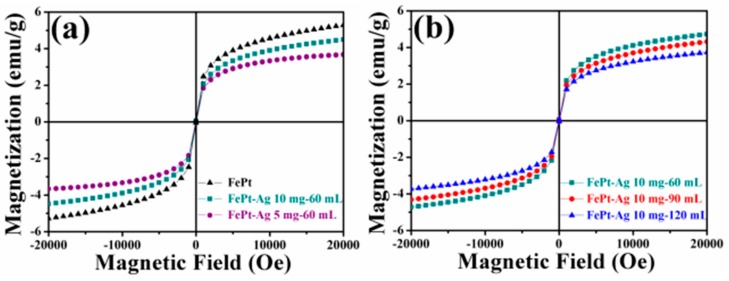
Magnetic hysteresis (*M-H*) loops of the pure FePt nanocrystals and the FePt–Ag nanocomposites with different additive quantities of FePt nanocrystals (FePt–Ag 5 mg–60 mL and FePt–Ag 10 mg–60 mL) (**a**), and the FePt–Ag nanocomposites with different additive amounts of silver colloids (FePt–Ag 10 mg–60 mL, FePt–Ag 10 mg–90 mL, and FePt–Ag 10 mg–120 mL) (**b**).

The magnetic properties of the pure FePt nanocrystals and the FePt–Ag nanocomposites were characterized by SQUID at room temperature. Figure 7 shows the magnetic hysteresis (*M-H*) loops of the pure FePt nanocrystals and the FePt–Ag nanocomposites. The magnetic saturation (*Ms*) of the pure FePt nanocrystals is 4.8 emu∙g^−1^. It is obvious that the *Ms* value decreases with the decrease of the addition quantities of FePt nanocrystals or the increase of the additive amount of the silver colloids. On the basis of the previous XRD, TEM, EDS, and XPS results, both the decrease of the addition quantities of FePt nanocrystals and the increase of the additive amount of the silver colloids may lead to the increase of Ag seeds in FePt–Ag nanocomposites. Therefore, the decrease in *Ms* values may be ascribed to the increase in weight ratio of Ag seeds to FePt nanocrystals [67] or the diamagnetic contribution of the intact Ag seeds [68]. It must also be mentioned that although *Ms* values decrease due to the introduction of Ag seeds, the FePt–Ag nanocomposites still have strong magnetic responsivity and can be easily magnetically separated from aqueous solution by using an external magnet (as shown in Figure S4), which is beneficial to their economic applications and reuse.

The temperature-dependent magnetization (*M-T*) of pure FePt and FePt–Ag 10 mg–120 mL was measured under zero-field-cooled (ZFC) and 1000 Oe field-cooled (FC) conditions (Figure S5). The shape of ZFC-FC curves for the two samples is the typical form for weakly dipole–dipole interacting systems. The magnetization of FC curves monotonically increases with the decrease of temperature. The ZFC curves reach maximum at 86 and 81 K for the pure FePt and FePt–Ag 10 mg–120 mL, respectively, corresponding to the blocking temperature (T_B_). In general, the T_B_ value is closely related to the nanocrystal size and the magnetic interaction between the nanocrystals [69]. It is reasonable to exclude the contribution of the FePt and Ag particle size to the variation of T_B_ value. Thus, the Ag seeds in the FePt–Ag nanocomposites weaken the magnetic dipole–dipole interaction between adjacent FePt nanocrystals, and result in decrease of the T_B_ value [70].

### 3.5. Catalytic Studies of FePt–Ag Nanocomposites

**Figure 8:**
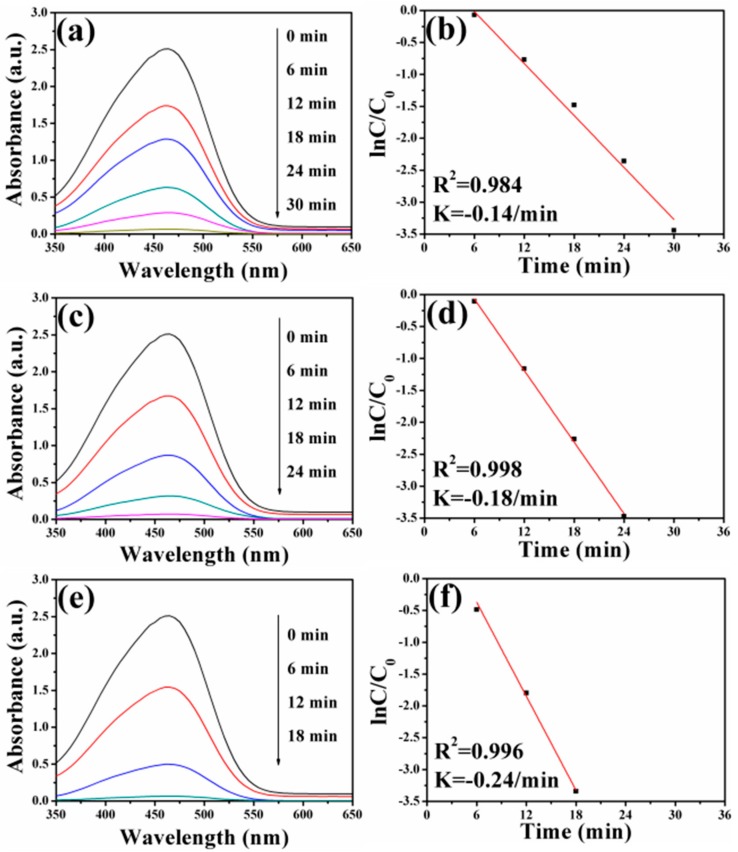
UV–vis absorption spectra of MO aqueous solution after reduction catalyzed by FePt–Ag 10 mg–60 mL (**a**), FePt–Ag 10 mg–90 mL (**c**), and FePt–Ag 10 mg–120 mL (**e**). (**b**,**d**,**f**) show the corresponding ln(*C*/*C*_0_) versus reaction time plots.

Previous works indicated that Ag nanocrystals played significant roles in the catalytic reduction of dye pollutants [71,72]. In order to study the catalytic performance of FePt–Ag nanocomposites on organic dyes, MO and RhB were selected as model dye pollutants. Figure 8 shows UV–vis absorption spectra of MO aqueous solution over time catalyzed by the different FePt–Ag nanocomposites (FePt–Ag 10 mg–60 mL, FePt–Ag 10 mg–90 mL and FePt–Ag 10 mg–120 mL) in the presence of NaBH_4_. As can be seen, the characteristic absorption peak of MO at 465 nm disappears within 30, 24, and 18 min of reaction time, respectively. This result indicates that the catalytic reduction and the complete removal of MO have been successfully achieved due to the inclusion of the FePt–Ag nanocomposites, which can be confirmed by the decoloration of MO in the inset of Figure 1. The corresponding ln(*C*/*C*_0_) versus reaction time plots are presented in Figure 8b,d,f. The concentration of MO at reaction time *t* is denoted by *C*, and the initial concentration of MO at *t* = 0 is regarded as *C*_0_. The concentration of NaBH_4_ can be considered as a constant during the reaction period, because it is much more excessive relative to that of MO. The ratio of *C* to *C*_0_ is obtained from the relative intensity of respective absorbance (*A_t_*/*A*_0_) at 465 nm. The linear relationship of ln(*C*/*C*_0_) versus reaction time shows that the reduction of MO by the FePt–Ag nanocomposites matches well with pseudo-first-order kinetics [73]. The rate constant is determined by a linear plot of ln(*C*/*C*_0_) versus reaction time. The correlation coefficients *R*^2^ of the two samples are higher than 0.98. The rate constants of MO are 0.14 min^−1^, 0.18 min^−1^, and 0.24 min^−1^ using FePt–Ag 10 mg–60 mL, FePt–Ag 10 mg–90 mL, and FePt–Ag 10 mg–120 mL as nanocatalysts, respectively. It is obvious that the rate constants for MO reduction increase with the increase of the additive amount of silver colloids. NaBH_4_ acts as the electron donor (BH_4_^−^ ions) and hydrogen supplier in this reaction [74]. When the FePt–Ag nanocomposites are added into the above reaction, the BH_4_^−^ ions and the MO molecules are absorbed on the surfaces of the FePt–Ag nanocomposites. The FePt–Ag nanocomposites can transfer the electrons from the donor BH_4_^−^ to the acceptor MO molecules and the BH_4_^−^ ions also transfer surface hydrogen to MO molecules. It is reasonable that if the noble metal nanomaterial loading is increased, the mass fraction of the noble metal nanomaterials is higher in the final samples, and, thus, a better catalytic performance should be accomplished [75].

**Figure 9:**
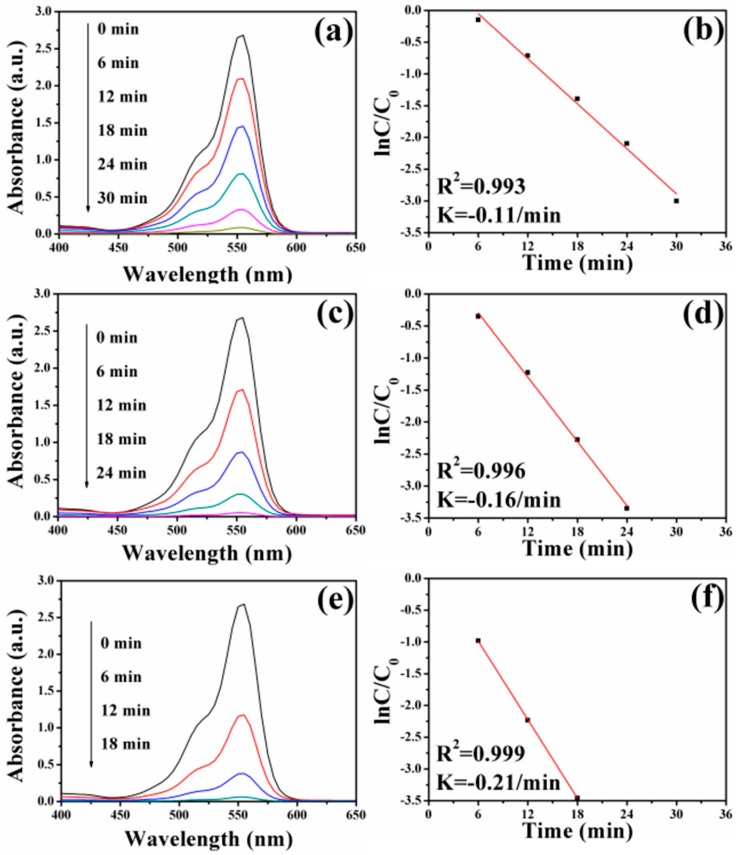
UV–vis absorption spectra of RhB aqueous solution after reduction catalyzed by FePt–Ag 10 mg–60 mL (**a**), FePt–Ag 10 mg–90 mL (**c**), and FePt–Ag 10 mg–120 mL (**e**). (**b**,**d**,**f**) show the corresponding ln(*C*/*C*_0_) versus reaction time plots.

In the case of RhB, a similar decolorization from pink to transparent is also observed in the insets of Figure 1. The changes in UV–vis absorption spectra of RhB aqueous solution over time catalyzed by the different FePt–Ag nanocomposites are shown in Figure 9. The intensity of the maximum absorption peak at 555 nm for RhB gradually decreases. In addition, an identical trend that the rate constants for RhB reduction increase when increasing the additive amount of silver colloids is found. It indicates that the catalytic ability of as-prepared FePt–Ag nanocomposites is not only efficient to a specific dye but also to different industrial dyes. Notably, as can be seen from Figures S6 and S7, no appreciable changes of the UV–vis absorption spectra for MO and RhB are observed when FePt nanocrystals or NaBH_4_ only are chosen as the catalysts, which suggests that MO and RhB could not be effectively reduced in the presence of FePt or NaBH_4_ only. Therefore, the catalytic reaction ability against dye pollutants mainly depends on the presence and the number of Ag seeds in the catalysts.

**Figure 10:**
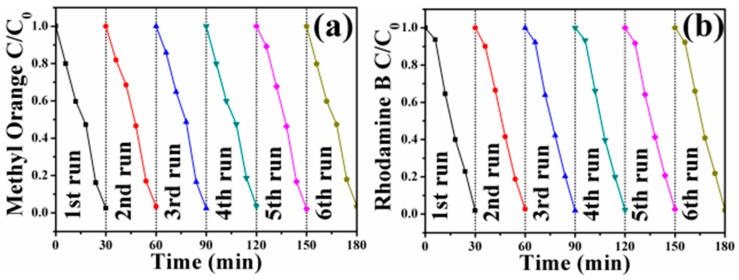
Six cycles of the removal of MO (**a**) and RhB (**b**) with FePt–Ag 10 mg–120 mL.

In order to investigate the recyclability of the FePt–Ag nanocomposites, FePt–Ag 10 mg–120 mL was selected to be separated magnetically and reused after catalytic reduction. As shown in Figure 10, FePt–Ag 10 mg–120 mL can be successfully recycled and reused against the two dye pollutants for at least six reaction runs with stable efficiency of around 100%, which indicates that the FePt–Ag nanocomposites have excellent reusability and can serve as recoverable and efficient catalysts for dye pollutants.

## 4. Conclusions

In summary, we have demonstrated the fabrication of pure FePt nanocrystals. Then, FePt–Ag nanocomposites were successfully synthesized through a novel seed deposition process. XRD, TEM, and SEM results indicate that the additive amount of silver colloids and FePt nanocrystals is essential to controlling the number of Ag seeds deposited on the PEI-DTC-modified surfaces of FePt nanocrystals. The increase of the number of Ag seeds attached to the FePt nanocrystals surfaces results in the shifts of Fe *2p* peaks and Pt *4f* peaks toward the lower binding energy side and shifts of Ag *3d* peaks toward the higher binding energy side. Although the *Ms* value decreases with the increase of the amount of Ag seeds attached on the FePt surfaces, the samples still have strong magnetic responsivity and can be easily separated from aqueous solution by applying a magnetic field. The pseudo-first-order kinetics are used to calculate the rate constant of MO and RhB. The catalytic reaction ability of FePt–Ag nanocomposites remains almost consistent even after at least six cycles, demonstrating that the FePt–Ag nanocomposites can serve as recyclable nanocatalysts for organic dyes. This system could potentially be applied as highly efficient nanocatalysts to different types of organic dyes.

## Supplementary Materials

Supplementary materials can be found at http://www.mdpi.com/2079-4991/8/5/329/s1. Figure S1: Particle size histogram of the pure FePt nanocrystal. Inset shows the corresponding TEM images of the pure FePt nanocrystals. Figure S2: Energy-dispersive spectroscopy (EDS) spectra of FePt (a), FePt–Ag 10 mg–60 mL (b), FePt–Ag 10 mg–90 mL (c), and FePt–Ag 10 mg–120 mL (d). Figure S3: XPS survey scan spectra of pure FePt nanocrystals and FePt–Ag 10 mg–120 mL nanocomposites. Figure S4: Photographs of the colloidal silver solution, FePt nanocrystals dispersed in deionized water, and FePt–Ag nanocomposites dispersed in deionized water and after gentle shaking. Figure S5: ZFC and FC curves of pure FePt nanocrystals and FePt–Ag 10 mg–120 mL nanocomposites under an applied field of 1000 Oe. Figure S6: UV–vis absorption spectra of MO aqueous solution (a) and RhB aqueous solution (b) after reduction catalyzed by pure FePt nanocrystals. Figure S7: UV–vis absorption spectra of MO (a) and RhB (b) reduced by NaBH_4_ only.
