# Supplementary material for: Highly Efficient, Low-Cost, and Magnetically Recoverable FePt–Ag Nanocatalysts: Towards Green Reduction of Organic Dyes
Source: Nanomaterials (Basel). 2018 May 14;8(5):329. doi: 10.3390/nano8050329 (PMC5977343; doi:10.3390/nano8050329)
Supplement: Supplementary file 1 [file nanomaterials-08-00329-s001.pdf]

Highly Efficient, Low-Cost, and Magnetically Recoverable FePt–Ag Nanocatalysts: Towards Green Reduction of Organic Dyes

Yang Liu [1,2,†], Yuanyuan Zhang [1,2], Qiangwei Kou [1,2], Yue Chen [1,2], Yantao Sun [2], Donglai Han [3,†], Dandan Wang [4], Ziyang Lu [5], Lei Chen [1,2], Jinghai Yang [1,2,*] and Scott Guozhong Xing [6,*]

[1] College of Physics, Jilin Normal University, Siping 136000, China; liuyang@jlnu.edu.cn (Y.L.); 13944139606@163.com (Y.Z.); 13944949603@163.com (Q.K.); 17649973053@163.com (Y.C.); chenlei@jlnu.edu.cn (L.C.)

[2] Key Laboratory of Functional Materials Physics and Chemistry of the Ministry of Education, Jilin Normal University, Changchun 130103, China; syt@jlnu.edu.cn

[3] School of Materials Science and Engineering, Changchun University of Science and Technology, Changchun 130022, China; dlhan_1015@cust.edu.cn

[4] Technology Development Department, GLOBALFOUNDRIES (Singapore) Pte. Ltd., 60 Woodlands Industrial Park D, Street 2, Singapore 738406, Singapore; DANDAN.WANG@globalfoundries.com

[5] School of Environment and Safety Engineering, Jiangsu University, Zhenjiang 212013, China; lzy@mail.ujs.edu.cn

[6] United Microelect Corp. Ltd., 3 Pasir Ris Dr 12, Singapore 519528, Singapore

* Correspondence: jhyang1@jlnu.edu.cn (J.Y.); Scott_Xing@UMC.com (S.G.X.); Tel./Fax: +86-434-329-4566

† These authors contributed equally to this work.

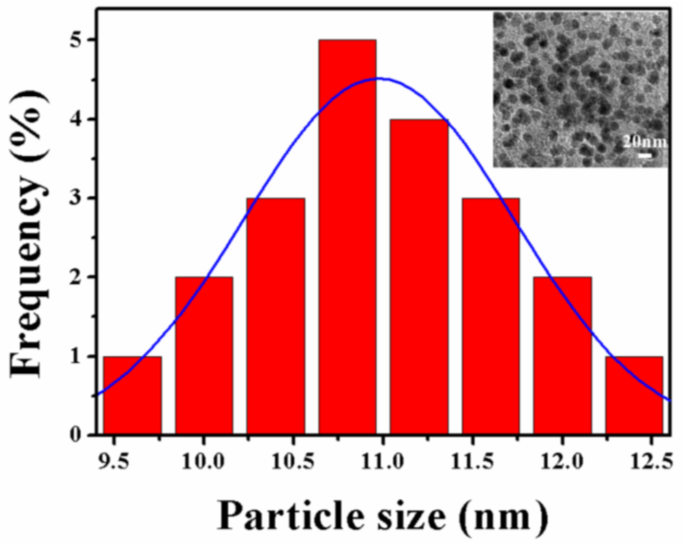

Figure S1. Histogram of the pure FePt nanocrystals particle size. Inset shows the corresponding TEM images of the pure FePt nanocrystals.

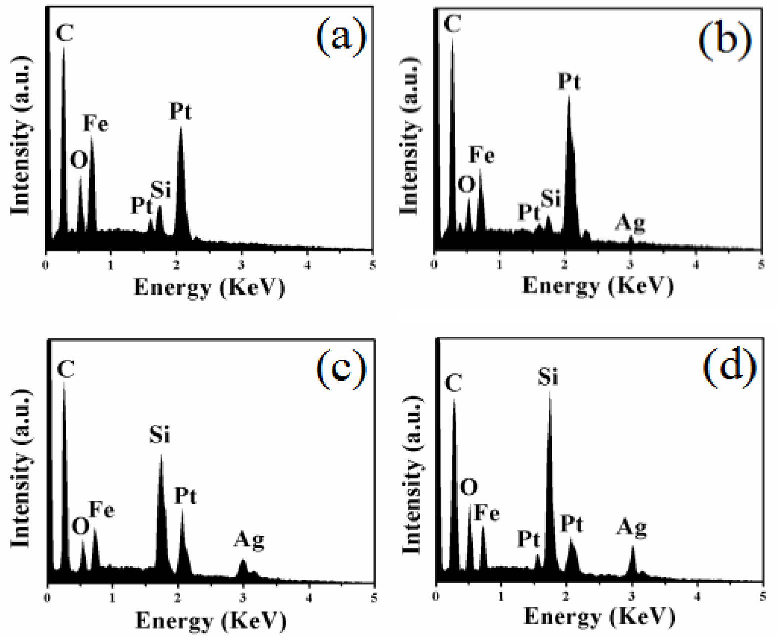

Figure S2. Energy-dispersive spectroscopy (EDS) spectra of FePt (**a**), FePt-Ag 10 mg–60 mL (**b**), FePt-Ag 10 mg–90 mL (**c**) and FePt-Ag 10 mg–120 mL (**d**).

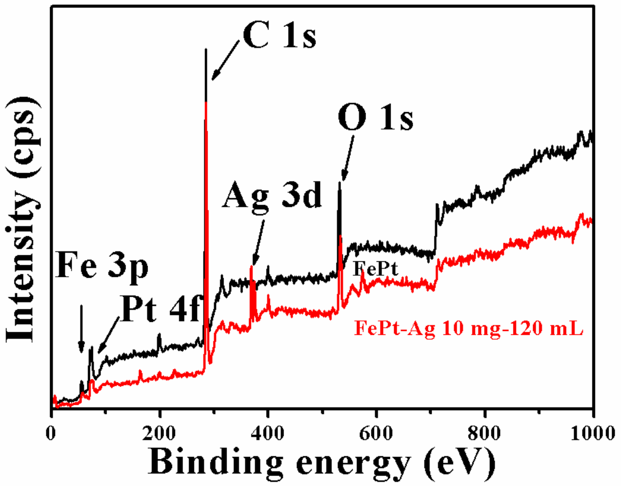

Figure S3. XPS survey scan spectra of pure FePt nanocrystals and FePt-Ag 10 mg–120 mL nanocomposites.

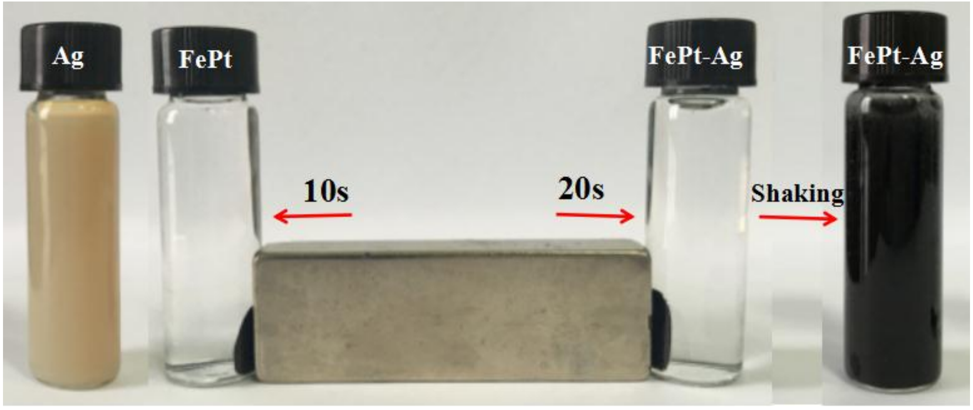

Figure S4. Photographs of the colloidal silver solution, FePt nanocrystals dispersed in deionized water, FePt-Ag nanocomposites dispersed in deionized water and after gentle shaking.

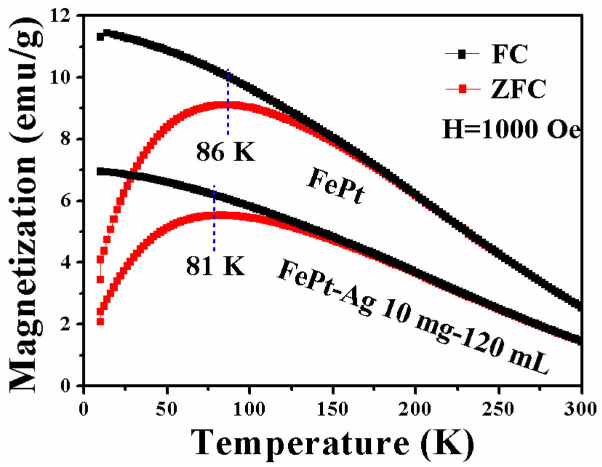

Figure S5. ZFC and FC curves of pure FePt nanocrystals and FePt-Ag 10 mg-120 mL nanocomposites under an applied field of 1000 Oe.

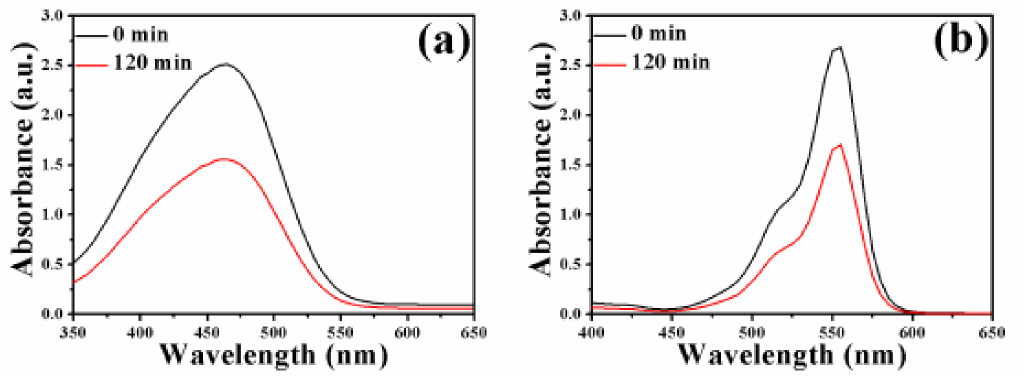

Figure S6. UV-Vis absorption spectra of MO aqueous solution (**a**) and RhB aqueous solution (**b**) after reduction catalyzed by pure FePt nanocrystals.

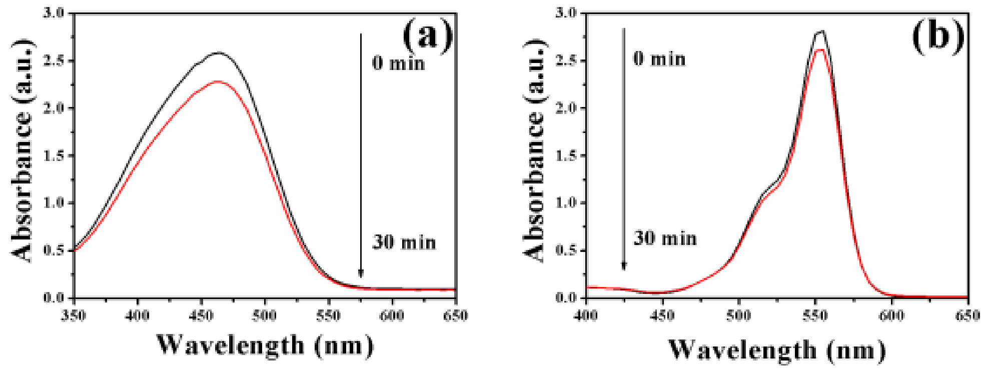

Figure S7. UV-Vis absorption spectra of MO (a) and RhB (b) reduced by $NaBH_4$ only.
